# A genome–wide screen to identify genes controlling the rate of entry into mitosis in fission yeast

**DOI:** 10.1080/15384101.2016.1242535

**Published:** 2016-10-13

**Authors:** Naomi Moris, Jaya Shrivastava, Linda Jeffery, Juan-Juan Li, Jacqueline Hayles, Paul Nurse

**Affiliations:** Cell Cycle Laboratory, The Francis Crick Institute, London, UK

**Keywords:** cell cycle, fission yeast, genomics, haploinsufficiency, rate-limiting, *S. pombe*

## Abstract

We have carried out a haploinsufficiency (HI) screen in fission yeast using heterozygous deletion diploid mutants of a genome-wide set of cell cycle genes to identify genes encoding products whose level determines the rate of progression through the cell cycle. Cell size at division was used as a measure of advancement or delay of the G2-M transition of rod-shaped fission yeast cells. We found that 13 mutants were significantly longer or shorter (greater than 10%) than control cells at cell division. These included mutants of the *cdc2, cdc25, wee1* and *pom1* genes, which have previously been shown to play a role in the timing of entry into mitosis, and which validate this approach. Seven of these genes are involved in regulation of the G2-M transition, 5 for nuclear transport and one for nucleotide metabolism. In addition we identified 4 more genes that were 8–10% longer or shorter than the control that also had roles in regulation of the G2-M transition or in nuclear transport. The genes identified here are all conserved in human cells, suggesting that this dataset will be useful as a basis for further studies to identify rate-limiting steps for progression through the cell cycle in other eukaryotes.

## Introduction

A more complete understanding of the eukaryotic cell cycle requires the global identification of gene functions necessary for cell cycle processes. To address this, near genome-wide gene deletion libraries constructed in yeast^1-4^ and RNAi approaches in Metazoa,^5-12^ have been used to identify genome wide sets of cell cycle genes. In fission yeast the rod-shaped cell and tip elongation growth pattern enables cell morphology to be used as an indicator of advancement or delay in progress through the cell cycle.^13-16^ This has allowed a comprehensive genome-wide visual screen of 4844 haploid gene deletion mutants (Bioneer library) of both essential and non-essential genes for elongated and small cell phenotypes typical of cell cycle mutants in fission yeast ^4^ and has identified 538 genes as being required for cell cycle progression. Deletion mutants of 513 genes were either delayed or blocked in cell cycle progression and had an elongated cell phenotype while a further 25 deletion mutants advanced cells through the cell cycle and showed a small cell phenotype.^1,4^

One of the key questions is which of these genes act as rate-limiting steps for cell cycle progression and so are involved in controlling this process. Genes giving a small cell phenotype when deleted are clearly candidates for contributing to control of the G2-M transition as absence of their gene function accelerates that transition.^17^ Another class of genes that are candidates for being rate-limiting at the G2-M transition are those which delay or block entry into mitosis if the function is partly compromised.^17^ In this study we use haploinsufficiency, exploiting the diploid heterozygous cell cycle gene deletion mutants to compromise gene expression, to identify regulatory genes that advance or delay cell cycle progression.

Screens for haploinsufficiency (HI), using mutants where one of two gene copies in a diploid strain has been deleted, provide a powerful way to identify gene products whose level is important for a biological process of interest.^1,17-20^ Rate-limiting steps within a regulatory network can impose limits on biological processes such as cell cycle progression, and such steps are potentially susceptible to a reduction in gene copy number. In budding yeast, heterozygous gene deletion diploid mutants usually show gene expression equivalent to half the level of the homozygous wild-type diploid.^21,22^ Therefore, screening heterozygous gene deletion diploid mutants for haploinsufficient genes is likely to identify rate-limiting steps for a process of interest. To identify genes that are HI for cell cycle progression we screened the heterozygous gene deletion diploid mutants of both essential and non-essential cell cycle genes for an increase or decrease in the cell size at septation, as a read-out of the timing at which cells enter mitosis.^14,17^ Mutants showing a delay or advancement into mitosis have previously identified components of the CDK regulatory network acting at the G2-M transition.^16,23^ CDK1-CyclinB activity (Cdc2-Cdc13 in fission yeast) is the key component of the mitotic control network and is regulated by Cdc2 tyrosine 15 phosphorylation by Wee1 kinase and dephosphorylation by Cdc25 phosphatase, respectively inhibiting or activating Cdc2 kinase activity.^24^ Cells enter mitosis when they reach a certain cell size; changing the levels of Wee1 or Cdc25 increases or decreases this size threshold, demonstrating that the activity of Cdc2 is a major rate-limiting step for cell cycle progression.^25,26^

## Results and discussion

**Figure 1.**
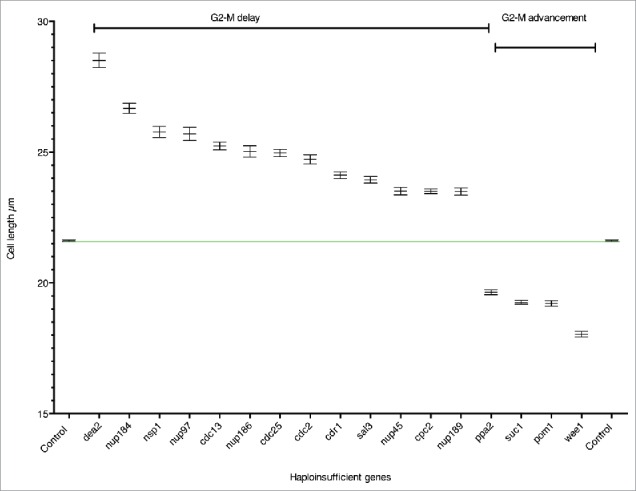
Haploinsufficient genes. Cell length at septation of heterozygous gene deletion mutants plotted as mean cell length with SEM. The green line shows the mean cell length of the control. n=>289 cells in at least 3 biological repeats.

We have carried out a haploinsufficiency screen of the genome wide set of cell cycle genes ^4^ to identify genes that are rate-limiting for cell cycle progression. Based on our initial results a further 31 genes, which did not have a typical cell cycle deletion phenotype, were also screened (see Table S1A column D, Materials and Methods for details). We measured cell length at septation for each of the diploid heterozygous gene deletion mutants and found a total of 85 mutants that showed a statistically significant deviation in cell length at septation from the control using Analysis of Variance (ANOVA) and Tukey post hoc tests (see Materials and Methods, Table S1B). The majority of these strains showed only small differences compared to the control. To focus on genes exhibiting the most significant effects we set a cut off in cell length at division of 10% longer or shorter than the control both for the mean and median values and identified 13 such genes (10% gene set) (Table 1, Fig. 1, Table S1B). Using esyN (http://www.esyn.org) ^27^ and the high confidence physical interaction data set from Pombase (http://www.pombase.org) together with GO slim term annotations these 13 genes were categorised into the following functional groups: i) Regulation of the G2-M transition (7 genes), ii) Nuclear cytoplasmic transport (5 genes) and iii) Nucleotide metabolism (one gene). Given that the HI group of genes included 4 previously identified HI genes *cdc2, cdc25, wee1* and *pom1*, all of which encode components of the rate-limiting CDK regulatory network,^17,28^ we concluded that other genes in this set are also likely to be important for the timing of the G2-M transition.

**Table 1.** Haploinsufficient gene set.

Gene	Gene E or V	% Deviation from control mean	% Deviation from control median	GO slim term biological process	Gene function
SPAC212.05c	V	0	0	control	Pseudogene
dea2	E	+31.94	+29.28	GO:0055086-nucleobase-containing small molecule metabolic process	adenine deaminase
nup184	V	+23.46	+23.43	GO:0006913 – nucleocytoplasmic transport GO:0006605 – protein targeting	nucleoporin
nsp1	E	+19.29	+18.17	GO:0006913 – nucleocytoplasmic transport GO:0042254 – ribosome biogenesis	nucleoporin
nup97	E	+18.92	+16.86	GO:0006913 – nucleocytoplasmic transport	nucleoporin Nic96 homolog
cdc13	E	+16.78	+16.72	GO:0007346 – regulation of mitotic cell cycle	G2/M B-type cyclin
nup186	E	+15.82	+13.34	GO:0006913 – nucleocytoplasmic transport	nucleoporin Nup186
cdc25	E	+15.55	+15.85	GO:0007346 – regulation of mitotic cell cycle	M phase inducer tyrosine phosphatase
cdc2	E	+14.43	+12.31	GO:0007346 – regulation of mitotic cell cycle	cyclin-dependent protein kinase
cdr1	V	+11.63	+11.80	GO:0007346 – regulation of mitotic cell cycle	NIM1 family serine/threonine protein kinase
sal3	V	+10.81	+10.87	GO:0006913 – nucleocytoplasmic transport	β importin
nup45	E	+8.80	+8.93	GO:0006913 – nucleocytoplasmic transport	nucleoporin
cpc2	V	+8.78	+8.72	GO:0002181 – cytoplasmic translation GO:0007010 – cytoskeleton organization	RACK1 ortholog
nup189	E	+8.74	+8.47	GO:0006913 – nucleocytoplasmic transport	nucleoporin Nup98 and Nup96
ppa2	V	−9.10	−8.94	GO:0007346 – regulation of mitotic cell cycle	serine/threonine protein phosphatase
suc1	E	−10.86	−10.90	GO:0000079 – regulation of cyclin- dependent protein serine/threonine kinase activity	cyclin-dependent protein kinase regulatory subunit
pom1	V	−11.06	−11.15	GO:0007010 – cytoskeleton organization GO:0007346 – regulation of mitotic cell cycle	DYRK family protein kinase
wee1	V	−16.50	−16.75	GO:0007346 – regulation of mitotic cell cycle	M phase inhibitor protein kinase

Heterozygous gene deletion diploid strains that divide at greater than 8% longer or shorter than the control strain for both mean cell length and median cell length. E= essential gene, V= non-essential gene.

**Figure 2.**
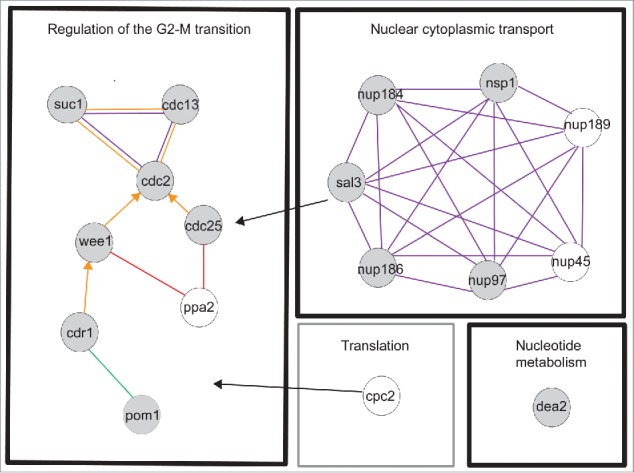
Functional groups and physical interactions for the HI genes. Evidence codes are orange edge = physical interaction, purple edge = within complex interaction, green edge = within pathway interaction, arrowhead = directed edge ie modification of a gene product by itself or another gene product, red edge = genetic interaction. Black arrows indicate genes involved in processes associated with more than one functional module. Gray filled circle = 10% gene set, unfilled circle = 8% gene set. The physical interactions were determined by esyN using Pombase high confidence interactions. The black boxes are the functional groups for the 10% gene set, the gray box is an additional functional group for the 8% gene set. This gene is also related to Regulation of the G2-M transition.

To ensure we did not miss any relevant genes just below the 10% cut-off, which were functionally related to the 10% gene set, we examined the biological function of genes 8–10% longer or shorter than the control at septation. Of the 7 genes in the 8–10% range (Table S1B) we identified 4 genes (8% gene set) that were greater than 8% longer or shorter than the control for both the mean and the median values and were directly related to Regulation of the G2-M Transition or Nuclear cytoplasmic transport (Table 1). We have considered both the 10% and 8% genes sets together as HI genes. The two categories Regulation of the G2-M transition and Nuclear cytoplasmic transport, included genes encoding products that physically interact in pathways or complexes (Fig. 2), further emphasizing the importance of these genes for cell cycle progression.

**Figure 3.**
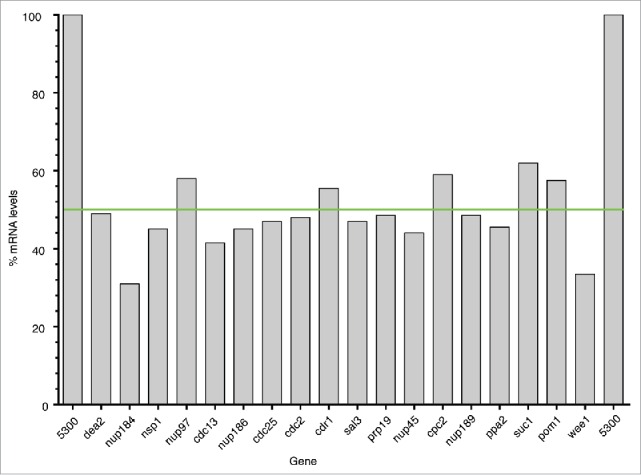
mRNA expression levels for the HI gene set. Graph showing the mRNA expression level for each the HI gene in the heterozygous deletion diploid strain compared to the homozygous control strain (100%) normalized to act1 mRNA. The green line denotes 50% of the control mRNA level.

All 17 strains were checked by PCR to confirm they were deleted for the correct gene, and all non-essential gene deletion mutants were checked to confirm that they had not become homozygous at the deletion locus (Table S1C, D). We carried out qPCR to estimate the mRNA levels encoded by a single copy of each HI gene, and found that the transcript level in all cases was reduced by around 50% (Table S1E, Fig. 3). This confirmed that for these genes there is no compensation by up-regulation of transcription from the remaining gene copy. For the 17 HI genes the mean and the median cell lengths were similar (Table S1B, compare columns H and K) and in the following discussion we have used the mean value when referring to cell length. The cell cycle gene set we used for the HI screen consisted of 368 (65.1%) essential genes and 197 (34.8%) non-essential genes (Table S1A). We found that the 17 HI genes had a similar distribution, with 10 (58.8%) essential genes and 7 (41.2%) non-essential genes (Fisher's Exact Test, Odds Ratio = 1.31, p > 0.05), suggesting that haploinsufficient cell cycle genes are no more likely to be essential or non-essential for cell cycle progression than the non-haploinsufficient genes.

We analyzed all 565 mutants using flow cytometric analysis. All mutants showed a 4C DNA content profile similar to the control; there was no evidence for a 2C or 2–4C population that would indicate a delay in G1 or S phase (data not shown). The failure to find any genes affecting progression through G1 or S phase may mean that gene products related to these processes are in excess, or perhaps more likely, our analysis may not have been sufficiently sensitive to detect the small delays or advances in G1 or S phase to be expected in a haploinsufficiency screen.

### The CDK1 mitotic control network

The timing of the G2-M transition is regulated by the conserved CDK1 mitotic control network ^24^ and we have identified 8 HI genes involved in this process (Table 1, Fig. 2). The cell length at septation of the 8 heterozygous deletion diploid strains ranged between +11.6% and +16.8% (*cdr1, cdc2, cdc25* and *cdc13*) and − 9.1% and −16.5% (*ppa2, suc1, pom1* and *wee1*) longer (+) or shorter (−) than the control (Table 1, Fig. 1, Table S1B). The HI genes *cdc2, cdc13, cdc25*, and *wee1* form the core of the mitotic control network.^24,29,30^ The Suc1 protein forms a complex with Cdc2 in fission yeast,^31^ and *suc1* orthologues in budding yeast and frogs have been shown to affect the phosphorylation levels of a subset of CDK1 substrates.^30,32, 33^ The fact that reduction of *suc1* gene dosage in fission yeast advances cells into mitosis suggests that Suc1 normally delays mitotic entry. It has previously been shown that when the *suc1* gene copy number in haploid cells is increased from one to two, cells are about 20% longer at cell division,^34^ supporting the idea that the level of Suc1 acts as a rate-limiting inhibitor for mitotic entry. The two genes, *pom1* and *cdr1* ^35-38^ both act in the Pom1 cell geometry pathway that regulates CDK1 activity by inhibiting Wee1 activity, while Ppa2 directly influences the CDK1 regulatory network through both Wee1 and Cdc25 (Table 1, Table S1B). Ppa2 is a subunit of the protein phosphatase PP2A, and loss of its activity in fission yeast causes cells to enter mitosis at a small cell size. In other organisms PPA2 has been shown to be part of a CDK1 autoregulatory feedback loop required for entry into mitosis^39,40^ and in fission yeast it is also involved in nutritional regulation of the G2-M size control.^41^

In addition to the genes within the mitotic control network, we identified 2 genes, *sal3* and *cpc2* that affect localization and translation efficiency of Cdc25 and Wee1 respectively. The *sal3* gene (+10.8%) is a β importin required for nuclear transport and plays a major role in Cdc25 nuclear localization, thus affecting the timing of the G2-M transition ^42^ (Fig. 2). The *cpc2* gene (+ 8.8%) encodes the fission yeast ortholog of mammalian RACK1 (Receptor for activated C kinase 1), a conserved ribosome associated protein with a central role in signaling.^43^ Cpc2 affects the efficient translation of a subset of proteins and may act as a scaffold for a number of signaling pathways in fission yeast.^44,45^ In the absence of Cpc2 the level of Wee1 is increased, while the level of the Wee1 inhibitor Cdr2 is decreased, suggesting that the observed increased cell length at division of both the haploid gene deletion and diploid heterozygous gene deletion mutants could be due to a delay in activation of the Cdc2 kinase at the G2-M transition.^46^ Cdr2 is a component of the Pom1 pathway and in our screen showed a statistically significant deviation in length at septation (+7.2%) to the control (Table S1B).

Previous studies, using reduction of function mutants of eIF4F subunits or the protein synthesis inhibitor cycloheximide, have also identified a link between translation efficiency and the translation of components of the CDK1 network; Cdc25, Wee1 and Cdc13.^47-51^ To see if any of these genes were HI for cell cycle progression we measured cell size at septation of the heterozygous gene deletion diploid mutants of eIF4A (SPAC1006.07), eIF4E (tif45), eIF4G (tif471) and the RNA helicase sum3/ded1/moc2. None of the 4 mutants showed a statistically significant deviation in cell length at septation from the control. This suggests that a reduction of gene copy number did not reduce gene function sufficiently to affect the translation efficiency of *cdc2, cdc13* or *cdc25*, and that these particular genes, although necessary for translation, are not HI for cell cycle progression. Regulating the translation efficiency of genes in the mitotic control network may work by directly regulating translation of genes regulating CDK activity or indirectly, for example through signaling pathways linking growth rate, monitored by translation efficiency, to the CDK network.

The 8 HI genes we have identified here all affect the timing of the G2-M transition and are rate-limiting for entry into mitosis. In addition Sal3 and Cpc2 are also potential candidates for linking other processes such as protein localization and translation efficiency to the regulation of the G2-M transition.

### Nuclear transport

Six HI genes encoded nucleoporins, *nup184* (+ 23.5%), *nup97* (+18.9%), *nsp1* (+19.3%) *nup186* (+15.8%) *nup45* (+ 8.8%) and *nup189* (+ 8.7%) (Table 1, Fig. 1, Fig. 3, Table S1B). The nuclear pore complex (NPC) consists of around 30 subunits and studies have shown that its basic structure is very similar in different organisms including fission yeast. There are 3 major groups of nucleoporins; membrane nucleoporins which link the NPC to the inner and outer nuclear membranes, scaffold nucleoporins that form the structure of the pore and FG (phenylalanine glycine) nucleoporins, which are required for transport selectivity.^52-54^ Five of the nucleoporins identified in this study, Nup186, Nup184, Nup97 (scaffold nucleoporins), Nsp1 and Nup45, (FG nucleoporins) are clustered together across the central core region of the nuclear pore.^53^ Nsp1, Nup97 and Nup45 are subunits of the Nic96 sub-complex identified in humans and budding yeast.^55^ This complex is required for nuclear pore assembly,^56^ and haploid fission yeast mutants deleted for either *nsp1* or *nup97* cells arrest as ungerminated spores, probably because a number of different cellular processes dependent on nuclear cytoplasmic transport are affected. However, when the gene dosage of either of these genes is reduced in diploid cells, cells are viable but show a cell cycle delay. Nup45 is also a Nic96 subunit, but unlike Nsp1 and Nup97, the *nup45* gene deletion mutant has a cell cycle phenotype in haploid cells as well as in the heterozygous gene deletion diploid mutant.^4,57^ The remaining Nic96 complex subunit Nup44 also has a cell cycle deletion phenotype in haploid cells and in our study showed a statistically significant deviation in cell length at septation (+7.1%) compared to the control (Table S1B). These data suggest that when the Nic96 complex is compromised, as in diploid heterozygous gene deletion mutants of *nsp1, nup97, nup45* or *nup44*, or in the haploid deletion mutants of *nup45* and *nup44*, it is primarily the cell cycle function that is affected.

The only nucleoporin we identified that is not found in the NPC central core was Nup189,^58^ which undergoes autocleavage to form 2 distinct nucleoporins (known as Nup189N or Nup98 and Nup189C or Nup96), although in fission yeast this cleavage is not essential for cell viability.^59^ Nup189N/Nup98 is an FG nucleoporin located in the outer ring of the nuclear pore, while Nup189C/Nup96 is a scaffold nucleoporin.

In HeLa cells, nucleoporins have been implicated in the G2-M transition as a knockdown of Nsp1/Nup62 (Nic96 subunit) causes a G2-M arrest,^60^ possibly as a result of altered localization of factors regulating the G2-M transition. It has also been shown that disassembly of the NPC at mitosis by CDK1 dependent phosphorylation of the GLFG repeats in Nup98 (Nup189N/Nup98 in *S. pombe*) is important for nuclear envelope breakdown and mitotic progression.^61^ Nucleoporins may also have roles that are independent of their role in nuclear transport. The Nic96 complex, for example, also affects chromosome segregation and spindle orientation^56,60^ and in budding yeast has a role ensuring equal segregation of nuclear pore complexes (NPCs) to daughter cells.^62,63^ Other studies in human cells have shown that nucleoporins may function as transcription factors regulating genes with a cell cycle related function.^64^ Nuclear transport also plays a central role during ribosome biogenesis,^65^ so it is possible that these haploinsufficient nucleoporins mainly affect translation and have an indirect effect on cell cycle progression.

All the nuclear transport genes identified in this screen are conserved in other eukaryotes ^66^ (http://www.pombase.org/ ), and as mentioned earlier, Sal3 (+10.8%) (Table 1, Fig. 1, Fig. 2) encodes a β importin regulating nuclear transport of Cdc25.^42^ Based on these results for Sal3 we propose that the nucleopore proteins identified here affect nuclear import or export of components of the CDK regulatory network.

### Other genes affecting the timing of entry into mitosis

The HI gene *dea2*, encodes an adenine deaminase (+31.9%)^67^ (Table 1, Figs. 1–3, Table S1B). Fission yeast adenine deaminases are related to the eukaryotic adenosine deaminases, which play a key role in the adenine salvage pathway (http://www.ebi.ac.uk/interpro/entry/IPR006330), and are involved in adenine catabolism.^67^ Because of its role in adenine catabolism in fission yeast a reduction in the level of Dea2 may lead to an imbalance between *de novo* synthesis, salvage and degradation of purines, which could affect both DNA replication and repair directly or indirectly via nucleotide/deoxynucleotide levels. A number of other genes involved in nucleotide metabolism (*adk1, hpt1, dut1, dcd1, tmp1*, and *dfr1*), although not haploinsufficient, have been identified as cell cycle genes,^4^ supporting the idea that levels of nucleotide intermediates are important for progress through the cell cycle. In HeLa cells the impairment of adenosine deaminase leads to high levels of dATP, which is an inhibitor of ribonucleotide reductase (RNR).^68,69^ In fission yeast the large subunit of RNR, encoded by *cdc22* and the small subunit encoded by *suc22*, are both essential for DNA replication and cell viability.^70^ However neither gene was identified in this study as HI either for the G2-M transition or progression through G1 or S phase (data not shown) nor did either gene show a statistically significant deviation in cell length at septation from the control. Flow cytometry showed that cells with reduced *dea2* gene dosage did not cause an obvious delay in G1 or S phase progression (Fig. S1), suggesting that the *dea2* heterozygous gene deletion strain is delayed in the G2 phase of the cell cycle. Perhaps when expression of *dea2* is only partially compromised (Fig. 3), RNR has sufficient activity to allow a doubling of DNA during S phase but that error prone DNA synthesis occurs due to increased levels of dATP, leading to activation of the DNA damage checkpoint and a delay of entry into mitosis. Alternatively S phase maybe extended with DNA replication continuing at a low level below our level of detection, due to reduced activity of RNR and activation of the DNA replication checkpoint to delay onset of mitosis.

### Rate-limiting steps

The idea of major rate-limiting steps controlling the rate of progress through a pathway was first postulated over 100 years ago.^71^ Since then work on metabolic control has led to the idea that many enzymes within a metabolic pathway contribute to flux through the pathway rather than there being a major step.^72^ For the rate of progression through the cell cycle, 2 major rate-limiting steps have been identified, one at the G1-S and one at the G2-M transition.^73-76^ For both of these transitions the key step is activation of CDK1 activity.^74,75,77^ In this study we have identified a number of genes that regulate Cdc2 activity, most of which act within the Cdc2 mitotic control network (Fig. 2). These results suggest that regulation of Cdc2 kinase activity by the mitotic control network occurs at a number of different steps each contributing to the overall timing of entry into mitosis. Other HI genes, for example *cpc2, dea2* and *sal3* affect other processes as well as cell cycle progression and these genes could be acting as major rate-limiting steps linking processes such as translation efficiency, nucleotide levels and localization of cell cycle regulators with cell cycle progression. Our results suggest that regulation of cell cycle progression depends not only on the major rate-limiting steps but is distributed further to a number of different steps, integrating multiple input signals feeding into the major cell cycle control genes.

Our haploinsufficiency screening approach has identified the canonical rate-limiting gene functions regulating the CDK1 mitotic control network involved in the G2 to mitosis transition, *cdc2, cdc13, wee1* and *cdc25*. In addition we have identified a further 4 genes, *suc1, cdr1, ppa2* and *pom1*, all previously shown to interact with components of the mitotic control network. Of these 4 genes, *suc1, cdr1* and *ppa2* have not previously been shown to be rate-limiting when their expression is reduced in a limited way. Translation efficiency has previously been implicated in cell cycle progression, and we have identified *cpc2* that influences translation efficiency and may be rate-limiting for levels of the CDK1 mitotic control network components. In addition we have identified 6 genes encoding nuclear pore complex subunits, Nup184, Nup186, Nup189N/Nup98, Nup189C/Nup96, Nsp1, Nup97, Nup45 and Sal3 a β importin which imports Cdc25 into the nucleus and regulates its nuclear localization.^42^ We propose that nuclear transport plays a key rate-limiting role in the regulation of the CDK1 mitotic control network, which has not been fully recognized. Finally we have identified Dea2 that, unexpectedly, implicates nucleotide metabolism in regulation of the G2-M transition, perhaps via a defect in DNA replication and activation of the DNA damage/replication checkpoint controls.

As the number of identified open reading frames increases in *S. pombe* (currently 5118 protein coding genes, http://www.pombase.org/status/statistics ), more genes important for cell cycle regulation can be expected to be identified. For example new open reading frames of less than 100 amino acids identified in fission yeast and deletion of genes not included in the Bioneer library have revealed a further 2 cell cycle genes.^78,79^ Further analysis of the Bioneer non-essential gene collection of ∼3000 haploid deletion mutants has also identified additional cell cycle genes. A screen for genes generating a small (wee) cell deletion phenotype identified 18 genes (an additional 8 genes).^80^ A multiprocess screen uncovered 25 genes in addition to the 538 genes previously identified, which affected the duration of the cell cycle phases but which did not have a long cell deletion phenotype, and a further 9 genes that when deleted result in an increased cell volume at cell division.^81^

As genes required for the cell cycle and its regulation are highly conserved in eukaryotes ^24^ it is likely that the haploinsufficient genes identified here as new rate-limiting components of the CDK1 mitotic network and nuclear transport, are also important for the timing of progression through the cell cycle in other eukaryotes. Further investigation of the role of these genes and the identification of new cell cycle genes in both fission yeast and other eukaryotes is likely to reveal new levels of cell cycle control and illuminate how this is linked to other cellular processes such as translation and cellular localization.

## Materials and methods

### Media and cell growth conditions

These were as described by Moreno, Klar and Nurse^82^ unless otherwise stated.

### Gene set and cell length measurements

All strains were grown in YES liquid media at 32°C to mid exponential growth (∼2 × 10^6^ − 8 × 10^6^ cells /ml). Live cells were examined using a Zeiss Axioskop 40 with a X63 Plan APOCHROMAT 1.4 oil immersion objective and photographed using a Zeiss Axiocam. Cell length for ∼30 septated cells for the initial analysis and at least 100 septated cells for each biological repeat was measured using pointpicker in Image J, (Table S1B). The subset of heterozygous diploid mutants used for this study was from the genome-wide set constructed by The South Korean consortium including Bioneer ^1^ and used by Hayles et al.^4^ The majority of strains in all the versions of the Bioneer haploid libraries are derived from this diploid heterozygous gene deletion library. The 17 HI strains identified in this study were the originally constructed diploids, except for 3 strains heterozygous for deletions of cdc25, wee1 or sal3, which were host exchanged diploids to remove a background ‘miss’ mutation.^1^ The working code suffix −0 or −1 (Table S1A column C) denotes host exchange or reconstructed deletion respectively.

Four genes from the genome wide, 538 gene set were omitted from the study for technical reasons e.g. strains were a mixed culture or haploidised at high frequency and we included 31 genes that when deleted do not have the classical cell cycle phenotype of long or small cells. These genes were included as they were known to be required for the cell cycle (4 genes), were related to genes we identified as HI genes (23 nuclear transport related genes) or were required for the initiation of translation (4 genes) (Table S1A column D).

### Flow cytometry analysis

Flow cytometry analysis was carried out as described in,^83^ using a Becton Dickinson FACScan.

### PCR and primer sequences

Total RNA was isolated from *S. pombe* cells using the hot phenol RNA extraction method^84^ and purified using RNeasy columns (Qiagen). Contaminating genomic DNA was removed by DNase 1 treatment (Ambion rDNase1). cDNA was synthesized using a Superscript III Invitrogen kit (Catalog no. 18080–051) with Oligo(dT)_20_ primers. qPCR reactions were carried out using EXPRESS SYBR® GreenER™ (Invitrogen) according to manufacturer's recommendations in an ABI 7500 Fast qPCR machine (annealing temp of 56°C). Levels of transcript were quantified using a standard curve method. Two biological repeats were carried out for each strain.

Primers used to confirm that the strain was deleted for the correct gene are shown in Table S1C and to confirm the strain was heterozygous for non-essential gene deletions are shown in Table S1D. Primers used for qPCR to measure the transcript level are shown in Table S1E. All primers were selected using ApE-A plasmid editor v1.17.

### Statistical analyses

Cell lengths from each mutant strain and wildtype measurements were analyzed using R software with the core statistics package (see Table S1B for summaries). The dataset was fitted with an Analysis of Variance (ANOVA) model, which identified significant differences between the strains (F = 29.54, df = 565, p <2e-16). To identify which strains contributed to this difference, a post-hoc Tukey test was applied in order to identify strains with significantly different cell length measurements from the control strain. To determine which genes could confidently be designated as HI we used 2 criteria: a) The distribution of cell length measurements showed a statistically significant deviation from the control cell length measurements and, b) The average cell length was ≥10% longer or ≤10% shorter than that of the wildtype control. The implementation of this second criterion was in order to minimise type I errors (false-positives) and ensure that all genes designated as HI were also likely to be biologically significant. While the 10% cut-off was somewhat arbitrary, the range is similar to the batch-variability of the wildtype strain (141 batches, median = 21.416 µm, lowest batch median = 19.11 µm (10.7% shorter), highest batch median = 23.53 µm (9.8% longer)). Additional genes functionally related to the HI genes are referred to within the text when they show average lengths ≥8% significantly longer or ≤8% significantly shorter than the control, in order to highlight similar behavior within classes of genes.

Batch effects between different experiments of the same strain were also tested by ANOVA to validate the reproducibility of data sets and are shown in Table S1, column W.

## Supplementary Material

1242535_Supplemental_Material.zip

## Abbreviations

HI: haploinsufficiency or haploinsufficient
CDK1: cyclin dependent kinase 1
RACK: Receptor for activated C kinase
nup: nucleoporin
NPC: nuclear pore complex
