# Supplementary material for: A genome–wide screen to identify genes controlling the rate of entry into mitosis in fission yeast
Source: Cell Cycle. 2016 Oct 13;15(22):3121–30. doi: 10.1080/15384101.2016.1242535 (PMC5134717; doi:10.1080/15384101.2016.1242535)
Supplement: 1242535_Supplemental_Material.zip [file kccy-15-22-1242535-s001.zip › 1242535_Supplemental Material/Figure S1 Moris et al[1].pptx]

## Slide 1
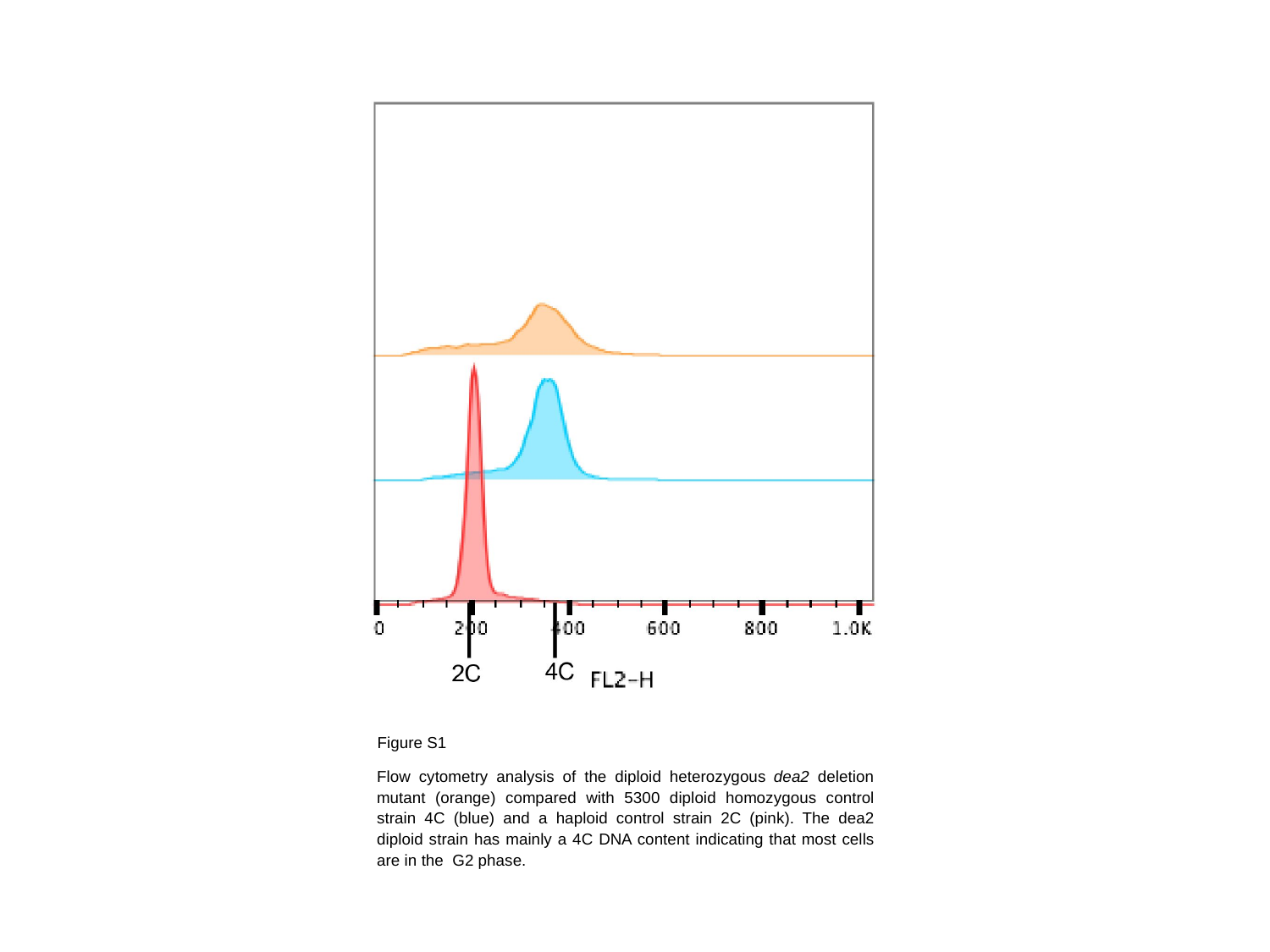

Figure S1
Flow cytometry analysis of the diploid heterozygous dea2 deletion mutant (orange) compared with 5300 diploid homozygous control strain 4C (blue) and a haploid control strain 2C (pink). The dea2 diploid strain has mainly a 4C DNA content indicating that most cells are in the G2 phase.
