# Supplementary material for: A genome–wide screen to identify genes controlling the rate of entry into mitosis in fission yeast
Source: Cell Cycle. 2016 Oct 13;15(22):3121–30. doi: 10.1080/15384101.2016.1242535 (PMC5134717; doi:10.1080/15384101.2016.1242535)
Supplement: 1242535_Supplemental_Material.zip [file kccy-15-22-1242535-s001.zip › 1242535_Supplemental Material/Table S1 legend Moris et al[1].docx]

**Table S1 legend**

**A**. Heterozygous gene deletion mutants used for the HI screen

**B**. A statistical analysis of 85 heterozygous gene deletion diploid strains that showed a significant different to cell length at septation compared to the control strain 5300 in which the KanMX gene replaced the pseudogene SPAC212.05c. ANOVA analysis of individual batches of cell length measurements showed that the *cdc13* heterozygous gene deletion strain showed significant batch differences. However the individual batches were all great than 10% longer than the control strain demonstrating that *cdc13* was an HI gene. The heterozygous diploid *snf21* deletion mutant although greater than 8% for both mean and median showed a significant difference between batches and so was excluded from the HI gene set (Table S1B column W). The bold black lines denotes the 10% and 8% gene sets

**C**. DNA primers for checking deletion of correct gene

Primers used to test that all genes greater than 8% longer/shorter than the control are deleted for the correct gene. Three methods were used; 1) 5’ gene specific primer CP5 + universal primer CPN1 or CPN10, 2) 3' gene specific primer CP3 + universal primer CPC1 or CPC3 and 3) sequencing of uptag with universal primers C10 and CPN10

**D**. Heterozygosity of gene deletion

Primers used to show that all heterozygous deletion mutants of non-essential genes that were 8% longer/shorter than the control had not homozygosed at the deletion locus to give a long/short cell phenotype. Two methods were used to check the presence of the wild type gene; 1) 5’ gene specific primer CP5 + wild type ORF specific primer or 2) wild type ORF specific primers

**E**. qPCR

Primers used to measure mRNA expression level of the wild type gene in the heterozygous deletion diploid strain of the 17 haploinsufficient genes using quantitative PCR
